# A mixture of ginger phenolic compounds enhances mitochondrial function, activates AMPK, and reduces lipid accumulation in adipocytes

**DOI:** 10.1371/journal.pone.0326690

**Published:** 2025-06-27

**Authors:** María Elizabeth Preciado-Ortiz, Berenice Pérez-Jiménez, Paulina Barrera-Gómez, Juan José Rivera-Valdés, Joshua Ayork Acevedo-Carabantes, Sarai Vásquez-Reyes, Armando R. Tovar, Nimbe Torres, Ivan Torre-Villalvazo, Erika Martínez-López

**Affiliations:** 1 Doctorado en Ciencias de la Nutrición Traslacional, Departamento de Clínicas de la Reproducción Humana, Crecimiento y Desarrollo Infantil, Centro Universitario de Ciencias de la Salud, Universidad de Guadalajara, Guadalajara, Jalisco, Mexico; 2 Instituto de Nutrigenética y Nutrigenómica Traslacional, Departamento de Biología Molecular y Genómica, Centro Universitario de Ciencias de la Salud, Universidad de Guadalajara, Guadalajara, Jalisco, Mexico; 3 Departamento de Fisiología de la Nutrición, Instituto Nacional de Ciencias Médicas y Nutrición Salvador Zubirán, Ciudad de México, Mexico; Universiti Putra Malaysia, MALAYSIA

## Abstract

Mitochondrial abundance and activity in adipocytes are critical for adequate adipose tissue function and whole-body energy homeostasis. Mitochondrial dysfunction in adipocytes impairs lipid metabolism, insulin sensitivity, and thermogenesis, leading to metabolic diseases. Enhancing mitochondrial function and density in adipose tissue may provide a promising therapeutic approach for metabolic diseases. This study evaluates the effects of a ginger phenol mixture on mitochondrial density and function, AMPK activation, lipid droplet content, and lipolysis markers in adipocytes differentiated *in vitro*. Pre-adipocytes isolated from the inguinal adipose tissue of Wistar rats were differentiated and assigned to three experimental groups: vehicle (0.2% DMSO), gingerol mixture (6 μg/mL), and positive control (1 mmol/m^3^ AMPK activator 5-Aminoimidazole-4-carboxamide ribonucleoside). Mitochondrial density and lipid content were assessed by MitoTracker and Bodipy staining respectively, while mitochondrial respiration was evaluated in an Extracellular Flux Analyzer. Protein abundance and basal lipolysis were evaluated by Western blotting and free fatty acids determination in supernatant, respectively. The gingerol mixture significantly enhanced mitochondrial density and respiration, including both maximal and ATP-linked capacities. Additionally, it activated AMPK, upregulated the expression of mitochondrial complexes, enhanced lipolysis markers, and reduced lipid droplet content. These findings suggest that the gingerol mixture enhances mitochondrial function, stimulates lipolysis, and reduces lipid accumulation in adipocytes, contributing to metabolic homeostasis in adipose tissue. This highlights its potential use as a complementary therapeutic agent for the management of obesity.

## Introduction

Adipose tissue serves as the primary energy reservoir in the body and plays a crucial role in the regulation of energy metabolism [1,2]. Under conditions of excess energy, adipose tissue stores the superabundant nutrients in the form of triglycerides. Conversely, during periods of energy scarcity, it supplies nutrients to other tissues through lipolysis, releasing free fatty acids [1–3].

Adipose tissue participates in the maintenance of whole-body energy homeostasis, and its function is significantly influenced by both the quantity and functionality of mitochondria [4,5]. Mitochondria generate cellular energy in the form of adenosine triphosphate (ATP) through glucose and lipid metabolism and produce several biosynthetic intermediates [6]. Furthermore, within adipose tissue, the activity of this organelle determines critical adipocyte functions including adipogenesis and thermogenesis. It also maintains insulin sensitivity, facilitates glucose and lipid metabolism, and participates in the crosstalk between adipose tissue and peripheral tissues, such as muscle and liver [4,7]. Conversely, mitochondrial dysfunction results in oxidative stress, cell death and inflammation in adipocytes, which promote detrimental effects on adipocyte differentiation, lipid metabolism, insulin sensitivity, oxidative capacity, and thermogenesis [6–8]. These alterations subsequently contribute to metabolic diseases such as insulin resistance, dyslipidemia, type 2 diabetes mellitus, arterial hypertension, and cardiovascular diseases [4,6].

Recent studies have suggested that enhancing mitochondrial function and increasing mitochondrial density in adipose tissue may represent a promising therapeutic strategy for the prevention and treatment of obesity and related metabolic disorders [5,7]. One key mechanism for stimulating mitochondrial biogenesis and functionality involves the activation of adenosine monophosphate-activated protein kinase (AMPK) [7]. Phosphorylation of AMPK promotes the activation of peroxisome proliferator-activated receptor gamma coactivator 1-alpha (PGC1α), which in turn enhances the expression of essential transcription factors for mitochondrial biogenesis and organelle functionality [9]. AMPK is activated by increased intracellular AMP levels, as seen during fasting and energy-demanding periods, such as exercise. Interestingly, AMPK can also be activated by small molecules, such as 5-Aminoimidazole-4-carboxamide ribonucleoside (AICAR), as well as bioactive food compounds [7].

Ginger (*Zingiber officinale Roscoe*) is widely recognized as a culinary spice and has been recommended for the treatment of obesity-related diseases [10]. The ginger root contains approximately 400 bioactive compounds, predominantly comprising gingerols, shogaols, and paradols [11]. In fresh rhizomes, the most abundant compounds are 6-, 8-, and 10-gingerol, while dried ginger primarily contains 6-, 8-, and 10-shogaols [12,13]. Studies in obese rats have demonstrated that administration of 6-gingerol leads to reductions in body weight and adipocyte size, downregulation of lipogenic and adipogenic gene expression, as well as improvements in insulin sensitivity and lipid profiles [14,15]. Furthermore, several in vitro studies using 3T3-L1 adipocytes have shown that various ginger-derived phenolic compounds modulate lipid metabolism by regulating key transcription factors involved in adipogenesis [16,17] and lipogenic enzymes [12,18,19]. Additionally, certain gingerols have been shown to regulate the activity of AMPK, which is associated with reduced fat accumulation and plays a role in mitigating endoplasmic reticulum stress [8,10].

However, the effect of a mixture of the main ginger phenols (6-gingerol, 8-gingerol, 10-gingerol, 6-shogaol, 8-shogaol, and 10-shogaol) on AMPK activation and mitochondrial function markers in adipocytes has not been thoroughly investigated. Therefore, the present study aimed to explore the impact of this ginger phenol mixture on mitochondrial density and function, AMPK activation, lipid droplet content, and lipolysis markers in adipocytes differentiated in vitro.

## Materials and methods

### Animals

Male albino rats *(Rattus norvegicus*, Wistar strain), weighing 150–200 g (6 months old), were obtained from the Experimental Research Department and Animal Care Facility at the Instituto Nacional de Ciencias Médicas y Nutrición Salvador Zubirán (DIEB-INCMNSZ). The rats were housed under a 12-h light/12-h dark cycle at 22 °C and had ad libitum access to standard laboratory chow. The experimental protocol was carried out in strict accordance with the recommendations in the Guide for the Care and Use of Laboratory Animals of the Committee for the Care and Use of Laboratory Animals (CICUAL) at the INCMNSZ. The protocol was approved by the CICUAL (Protocol Number: CICUAL-FNU-2141-25-26-1). Adipose tissue collection was performed after euthanasia by sevoflurane overdose, and all efforts were made to minimize suffering.

### Pre-adipocyte isolation and adipocyte differentiation

**Fig 1:**
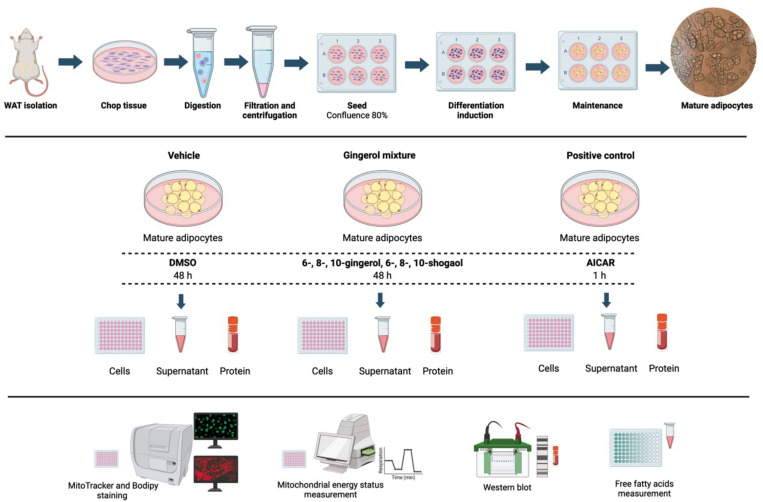
Experimental design. Primary cell culture of adipocytes was derived from the stromal vascular fraction (SVF) of the inguinal adipose tissue from Wistar rats. Mature adipocytes were divided into three experimental conditions: a vehicle control (DMSO), a gingerol mixture (comprising 6-, 8-, and 10-gingerol, 6-, 8-, and 10-shogaol) and a positive control (AICAR). In all groups, cells were stained with MitoTracker and Bodipy to assess mitochondrial density and lipid content. A mitochondrial stress assay was conducted to determine mitochondrial respiration. Protein abundance was analyzed using Western blotting, and the supernatant was collected to measure the content of free fatty acids.

The experimental design for the primary cell culture is illustrated in Fig 1. Pre-adipocytes were isolated from the stromal vascular fraction (SVF) of the inguinal adipose tissue collected from two male Wistar rats. After euthanasia by sevoflurane overdose, adipose tissue was washed twice with phosphate-buffered saline (PBS) to remove red blood cells and tissue debris. Then, it was fragmented into small pieces and digested using 0.5% type IV collagenase (Gibco Life Technologies, Grand Island, NY, USA) at 37 °C for 20 min with gentle shaking. After digestion, the cells were centrifuged at 700 × g for 5 min, and the supernatant was discarded. The SVF pellet was resuspended in PBS and filtered through a 100-μm cell strainer (Corning Inc., Corning, NY, USA). The suspension was centrifuged again at 700 × g for 5 min, and the SVF pellet was resuspended in DMEM/F-12 (Gibco Life Technologies, Grand Island, NY, USA). The SVF cells were then seeded in a flask containing complete medium, which consisted of DMEM/F-12 supplemented with 10% fetal bovine serum (Gibco Life Technologies, Grand Island, NY, USA) and 1% antibiotic/antimycotic mixture (Caisson Labs, Smithfield, UT, USA), and maintained at 37 °C in a 5% CO2 environment.

For adipocyte differentiation, the pre-adipocytes from the SVF were seeded in 6-well plates (Corning Inc, Somerville, MA, USA), and the medium was replaced every 48 h until the cells reached 80% confluence. Upon reaching confluence, cells were differentiated in complete medium supplemented with 1 mmol/m^3^ dexamethasone (Sigma-Aldrich Merck, Darmstadt, Germany), 1 mmol/m^3^ insulin (Sigma-Aldrich Merck, Darmstadt, Germany), 0.5 mol/m^3^ isobutylmethylxanthine (Sigma- Aldrich Merck, Darmstadt, Germany), and 1 mmol/m^3^ rosiglitazone (Sigma-Aldrich Merck, Darmstadt, Germany). After 72 h of differentiation, the medium was replaced with maturation medium, which contained complete medium with 1 mmol/m^3^ insulin (Sigma- Aldrich Merck, Darmstadt, Germany). The maturation medium was changed every 48 h for an additional 5 d. The total duration of adipogenesis was 8 d, starting from the initiation of differentiation.

### Mixture of ginger phenolic compounds

A mixture of gingerols and shogaols was obtained from Merck KGaA (cat. no. SIG-G-027-1ML). This solution contained the main phenolic compounds in ginger. The ginger phenolic compounds mixture was prepared by combining 500 μg/mL each of 6-, 8-, and 10-gingerol and 6-, 8-, and 10-shogaol, resulting in a 1:1 (w/v) ratio of gingerols to shogaols (gingerol mixture). Dilutions were prepared to achieve a final concentration of 6 μg/mL in dimethyl sulfoxide (DMSO; Sigma‑Aldrich; Merck KGaA; cat. no. 276855‑1L).

### Adipocyte treatment

Differentiated adipocytes were divided into three experimental conditions: Vehicle, treated with 0.2% DMSO for 48 h; Gingerol mixture, treated with 6 μg/mL gingerol mixture for 48 h and a positive control, treated with 1 mol/m^3^ AICAR for 60 min. Each condition was subjected to testing in three technical replicates (wells per condition), and the entirety of the experiment was conducted twice utilising cells obtained from individual rats (biological replicates) (Fig 1).

### Mitochondrial energy status measurement

Mitochondrial respiration in differentiated adipocytes was assessed using the Seahorse XF Cell Mito Stress Test Kit (Agilent, Santa Clara CA, USA) with the XFe96 Extracellular Flux Analyzer (Agilent Technologies, Santa Clara CA, USA).

After 72 h of differentiation, adipocytes were trypsinised and plated in XFe96 microplates (Agilent Technologies, Santa Clara CA, USA) at a density of 15,000 cells per well and maintained in maturation medium for an additional 5 d and treated according to their designated experimental conditions (Vehicle, Gingerol mixture, Positive control).

To measure mitochondrial energy status, cells were washed with XF basal medium (Agilent Technologies, Santa Clara, CA, USA) supplemented with 11 mol/m^3^ glucose, 1 mol/m^3^ pyruvate, and 2 mol/m^3^ glutamine. The cells were then incubated for 1 h in a CO2-free incubator with the same medium. Mitochondrial respiration was assessed by sequentially injecting the following compounds: 2 mmol/m^3^ oligomycin, 1 mmol/m^3^ carbonyl cyanide-p-trifluoromethoxy phenylhydrazone (FCCP), and 1 mmol/m^3^ rotenone/antimycin A (Agilent Technologies, Santa Clara, CA, USA). Oxygen consumption rate (OCR) measurements were obtained and analyzed according to the manufacturer’s recommendations (Seahorse Bioscience, Agilent Technologies, Santa Clara, CA, USA).

OCR and ECAR were normalized by in situ cell counts using a BioTek Cytation 5 as follows: Immediately following XF analysis, cells were fixed using 4% paraformaldehyde and then stained with 4′,6-diamidino-2-phenylindo (DAPI, Sigma-Aldrich). Cell images were captured using a 4x lens on the Cytation 1 (Agilent BioTek). The nuclear number was counted using the Cell Analysis function in the Gen5 software program, and data were exported to normalize XF data. All experiments were conducted in quintuplicate.

### Bodipy and MitoTracker staining

Adipocyte differentiation efficiency and total cell count were assessed using Bodipy (Invitrogen, Thermo Fisher Scientific, Waltham, MA, USA) and Hoechst (Thermo Fisher Scientific, Waltham, MA, USA) staining. For mitochondrial visualization, cells were stained with MitoTracker Deep Red FM (Thermo Fisher Scientific, Waltham, MA, USA). Adipocytes were incubated with 250 μmol/m^3^ Bodipy, 2 mmol/m^3^ Hoechst, and 50 μmol/m^3^ MitoTracker Deep Red FM at 37 °C for 30 min. Fluorescence was quantified using the Cytation 1 Cell Imaging Multi-Mode Reader (BioTek, Winooski, VT, USA). The number of adipocytes was determined by calculating the ratio of differentiated adipocytes to the total number of precursor cells for each condition.

### Western blot analysis

At the end of the treatments, adipocytes were harvested by washing the cells with PBS buffer and subsequently lysed in RIPA buffer containing PBS, SDS, sodium deoxycholate, sodium azide, NP-40, and protease and phosphatase inhibitors. Total protein was obtained by centrifugation at 15,000 x g for 20 min at 4˚C. Total proteins were separated by SDS-polyacrylamide gel electrophoresis and transferred to polyvinylidene difluoride membranes (Bio-Rad Laboratories, Hercules, CA, USA). Membranes were incubated overnight at 4°C with specific antibodies against AMPK, p-AMPK, p-HSL (1:1,000; Santa Cruz Biotechnology, Inc), and oxidative phosphorylation complexes (OXPHOS) (1:1000; Abcam, Cambridge, UK). Detection was achieved using anti-rabbit or anti-mouse secondary antibodies (1:20,000; Abcam, Cambridge, UK). The GAPDH antibody (1:40,000; Abcam, Cambridge, UK) was used as a loading control. Bands were visualized using Immobilon Western chemiluminescent HRP substrate (Millipore, Temecula, CA, USA). Chemiluminescence was digitized with the ChemiDoc MP imaging system (Bio Rad Laboratories, Hercules, CA) and analyzed with ImageJ 1.51 (100) 2015 software (NIH, USA).

### Free fatty acids measurement

Free fatty acids in the supernatants were quantified using the Free Fatty Acids Half Micro Test (Roche, St. Louis, MO, USA) according to the manufacturer’s instructions. All experiments were performed in triplicate.

### Statistical analysis

Data are presented as means ± standard error of the mean (SEM) to represent the precision of the estimated mean across replicate wells (n = 6). Statistical analyses were performed using one-way ANOVA followed by Tukey’s post hoc test to assess differences among group means. All analyses were conducted using GraphPad Prism 9.0 (GraphPad, San Diego, CA, USA), and differences were considered statistically significant at p < 0.05.

## Results

### Effects of a gingerol mixture on mitochondrial function in adipocytes

**Fig 2:**
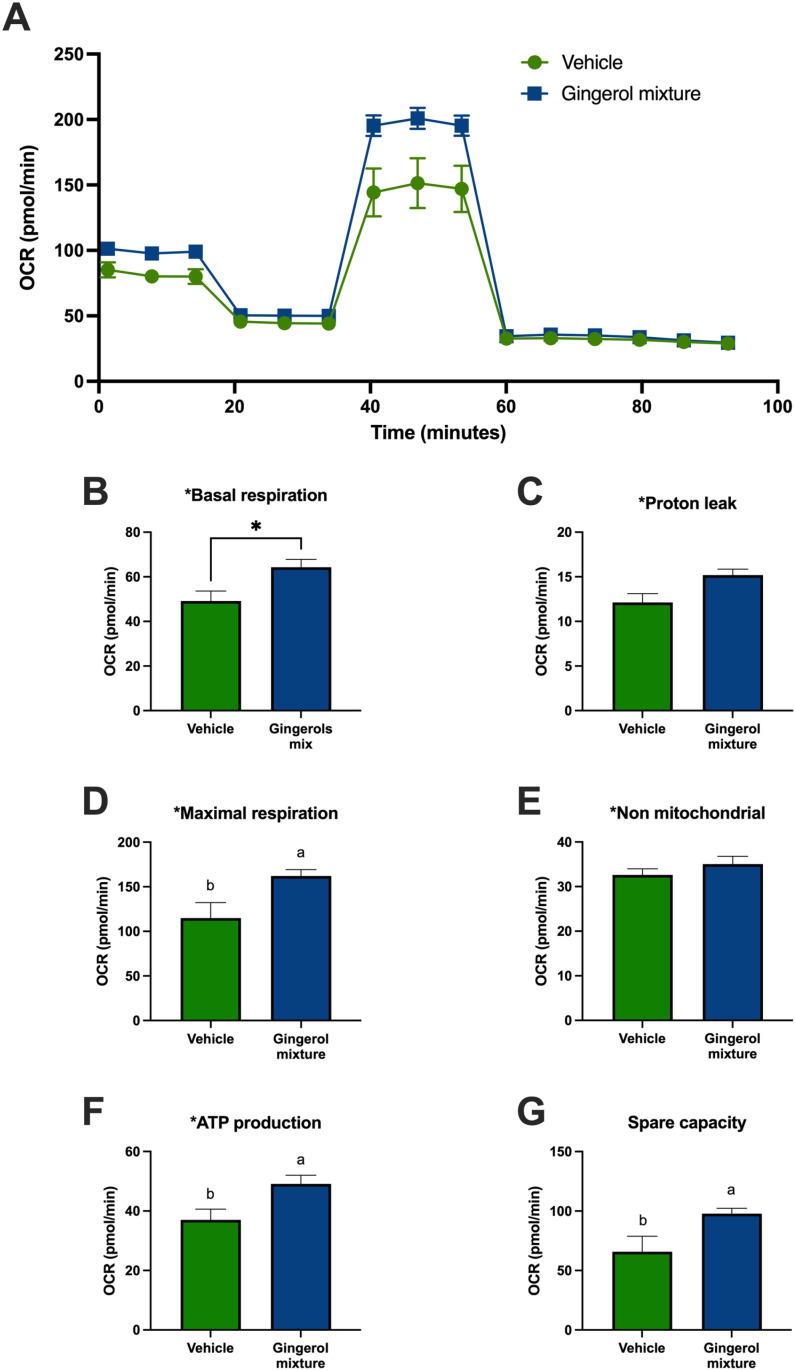
Oxygen consumption rate (OCR) and the calculated mitochondrial parameters of adipocytes treated with either a gingerol mixture or DMSO (vehicle) for 48 h. **(A)** OCR of adipocytes, **(B)** Basal respiration **(C)** Proton leak, **(D)** Maximal respiration, **(E)** Non-mitochondrial respiration, **(F)** ATP production, and **(G)** Spare capacity. Results are expressed as mean ± SEM, with assays performed in quintuplicate. Differences among groups were analyzed using one-way ANOVA followed by Tukey’s post-hoc test. Letters indicate significant differences between groups (a < b), P < 0.05.

To investigate the effect of the main ginger phenols on mitochondrial function and energy production in adipose tissue, the OCR in adipocytes was assessed using a mitochondrial stress test. SVF cells derived from inguinal adipose tissue were cultured and differentiated into adipocytes. Once fully differentiated, the adipocytes were treated with 6 μg/mL of a gingerol mixture or DMSO (as a vehicle control) for 48 h. As shown in Fig 2, the gingerol mixture significantly enhanced maximal mitochondrial respiration, ATP-linked respiration, and reserve respiratory capacity, without affecting other mitochondrial parameters, such as proton leak or non-mitochondrial respiration. These findings suggest that the predominant phenolic compounds in ginger root enhance mitochondrial activity in mature adipocytes.

### The gingerol mixture increased mitochondrial abundance, promoted AMPK activation, and OXPHOS expression in adipocytes

**Fig 3:**
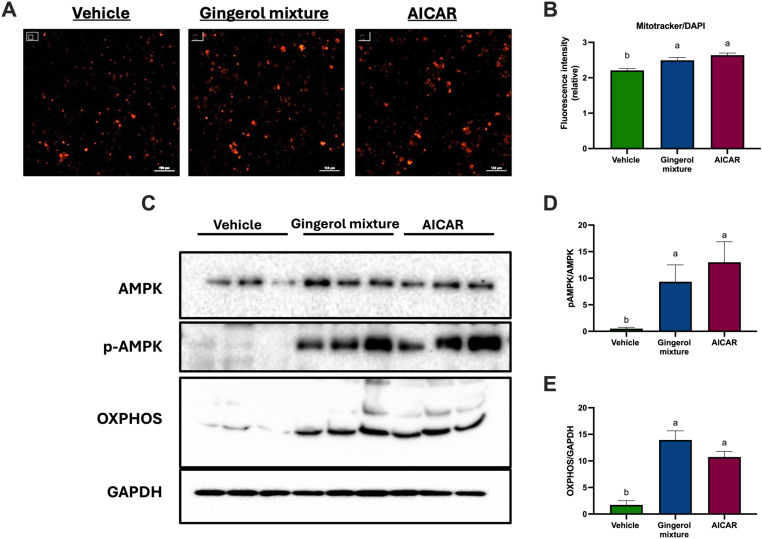
Effects of a gingerol mixture on mitochondrial abundance, AMPK phosphorylation and the abundance of mitochondrial protein complexes in adipocytes treated for 48 h. **(A)** MitoTracker fluorescence staining. **(B)** Relative fluorescence intensity of MitoTracker. **(C)** Immunoblot analysis of total AMPK, phospho-AMPK (p-AMPK), and oxidative phosphorylation complexes (OXPHOS). **(D)** Densitometric analysis of the pAMPK/AMPK ratio. **(E)** OXPHOS abundance in adipocytes. GAPDH was used as a loading control. The results are presented as mean ± SEM, with assays performed in triplicate. Differences among groups were analyzed using one-way ANOVA followed by Tukey’s post-hoc test. Letters indicate significant differences between groups **(a < b)**, P < 0.05.

To assess the effect of the gingerol mixture on mitochondrial density in adipocytes, the fluorescent dye MitoTracker was used. AICAR was used as a positive control, while DMSO served as the vehicle control. As expected, stimulation with AICAR significantly increased mitochondrial density compared to the vehicle. Notably, adipocytes treated with the gingerol mixture exhibited a higher mitochondrial density compared to the vehicle, with results similar to those observed in the positive control (Fig 3A, 3B).

Given that mitochondrial activity and abundance are partially regulated through AMPK activation, we investigated whether the increase induced by the gingerol mixture was mediated by AMPK activation. As shown in Fig 3C and 3D, treatment with AICAR resulted in increased AMPK phosphorylation, as expected. Importantly, the gingerol mixture also significantly enhanced AMPK phosphorylation compared to the vehicle, reaching levels comparable to those of the positive control.

Since AMPK signaling promotes mitochondrial biogenesis, we evaluated the abundance of proteins from mitochondrial complexes I, II, III, IV, and ATP synthase (collectively referred to as OXPHOS) in adipocytes using Western blot analysis. As observed in Fig 3C and 3E, the gingerol mixture increased the enzymatic components of the respiratory chain in adipocytes to levels similar to those observed with the positive control. These results indicate that the gingerol mixture enhances the abundance of mitochondrial complexes in adipocytes through AMPK activation.

### A gingerol mixture reduced adipocyte lipid droplet content and promotes lipolysis in adipocytes

**Fig 4:**
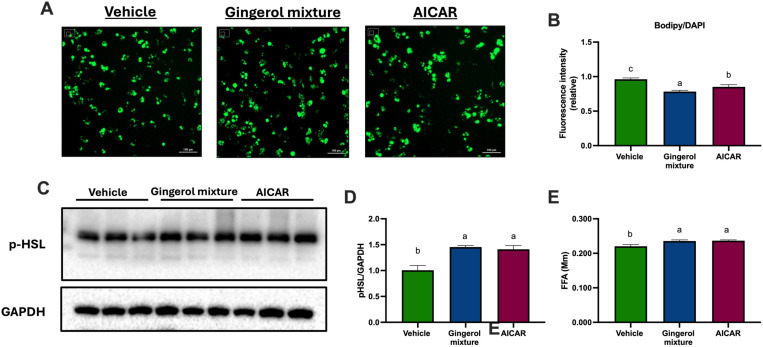
Effect of the gingerol mixture on lipid accumulation and lipolysis in adipocytes treated for 48 h. **(A)** Bodipy fluorescence staining. **(B)** Relative fluorescence intensity of Bodipy. **(C)** Immunoblot analysis of phospho-HSL (p-HSL). **(D)** Densitometric analysis of p-HSL. GAPDH was used as the loading control. **(E)** Measurement of free fatty acids released into the culture medium. Results are presented as means ± SEM, with assays performed in triplicate. Differences among groups were analyzed using one-way ANOVA followed by Tukey’s post-hoc test. Letters indicate significant differences between groups (a < b < c), P < 0.05.

To evaluate whether increased mitochondrial function leads to a reduction in lipid content in adipocytes, we assessed lipid droplet abundance using the fluorescent lipid dye Bodipy (Fig 4A, 4B). Adipocytes treated with the gingerol mixture exhibited fewer lipid droplets compared to both the vehicle and the positive control. These findings suggest that the gingerol mixture enhances fatty acid oxidation in adipocytes, thereby preventing excessive lipid accumulation.

In addition to the observed increase in mitochondrial fatty acid oxidation, the reduction in lipid droplet content in adipocytes treated with ginger phenols may also be attributed to enhanced lipolysis. Thus, we measured the abundance of phospho-HSL and the release of free fatty acids into the culture medium. As shown in Fig 4C and D, the gingerol mixture significantly increased HSL phosphorylation and, consequently, the abundance of free fatty acids in the culture medium compared to the vehicle, reaching levels comparable to those observed with AICAR. These results clearly demonstrate that AMPK activation by the gingerol mixture promotes HSL-dependent lipolysis in adipocytes.

## Discussion

The present study demonstrates that a gingerol mixture (comprising 6-, 8-, and 10-gingerol, as well as 6-, 8-, and 10-shogaol) enhances mitochondrial function and density, and promotes lipolysis in adipocytes, thereby reducing lipid accumulation. These effects were mediated by an increase in AMPK activity, which promotes mitochondrial biogenesis and elevated expression of mitochondrial machinery.

Adipocyte dysfunction, characterized by impaired mitochondrial function, oxidative stress and inflammation, plays a pivotal role in the early development of metabolic disturbances associated with obesity [7,8]. Recent studies have suggested that plant-derived bioactive compounds, such as gingerols, may have therapeutic potential in addressing these disturbances due to their effects on lipid metabolism, mitochondrial function, and anti-inflammatory properties [20]. In particular, these beneficial effects are closely related to mitochondrial functionality in adipose tissue [5,7].

Mitochondria are essential organelles involved in energy production and cellular oxidative metabolism, generating approximately 90% of cellular ATP through oxidative phosphorylation complexes (OXPHOS) [21]. The present study revealed that the gingerol mixture significantly enhanced mitochondrial function, as evidenced by increased maximal respiration, ATP-linked respiration, and reserve respiratory capacity. Additionally, the expression of OXPHOS machinery was upregulated, indicative of improved mitochondrial efficiency. These results are consistent with previous studies on capsaicin [22] and genistein [23] which have been shown to enhance mitochondrial respiration and energy expenditure in adipocytes [22,23]. Furthermore, this study demonstrated that the gingerol mixture increased mitochondrial abundance in adipocytes, corroborating findings from research on other polyphenols, such as quercetin and genistein, which have also been shown to promote mitochondrial biogenesis in 3T3-L1 cells [23,24].

Gingerols are well-known for their anti-obesity properties, particularly through their ability to modulate adipogenesis and lipid metabolism. Previous studies have shown that gingerol compounds reduce intracellular lipid accumulation and regulate the expression of key lipogenic genes in 3T3-L1 pre-adipocytes [16,18]. Consistent with these findings, this study demonstrated that treatment with a gingerol mixture significantly reduced lipid content in mature adipocytes. This reduction was accompanied by increased levels of free fatty acids in the supernatant, suggesting enhanced lipolysis. Moreover, the gingerol mixture increased phosphorylated hormone-sensitive lipase (p-HSL), a key regulator of lipolysis, further confirming its role in lipid breakdown [25].

The improvement in mitochondrial density and function, as well as reduction in lipid content and enhanced lipolysis observed in our study may be associated with the activation of AMPK, a central regulator of energy homeostasis. AMPK phosphorylation is known to promote mitochondrial biogenesis and enhance lipid catabolism [8,13]. Emerging evidence highlights the metabolic and vascular protective effects of ginger phenols, particularly 6- and 10-gingerol, by the activation of AMPK in various tissues, including adipose tissue [8,10]. In vascular injury models, 10-gingerol significantly reduced neointimal hyperplasia following carotid artery ligation via AMPK binding [10]. Similarly, 6-gingerol has shown robust activity in metabolic disease models: it improved insulin sensitivity, reduced hepatic steatosis and lipid accumulation, and alleviated inflammation and oxidative stress in high-fat diet-fed mice and palmitate-treated HepG2 cells [26,27]. In skeletal muscle and liver, 6-gingerol decreased lipid deposition and reactive oxygen species (ROS) production, while enhancing mitochondrial membrane potential and respiratory capacity via AMPK/SIRT1/PGC-1α signaling [28,29]. It also improved plasma metabolic profiles and adiponectin levels in female mice, and activated the AMPK/PGC-1α pathway in adipose tissue of aging rats [30]. In the present study, the effect of the gingerol mixture on mitochondrial functionality and the reduction in lipid accumulation was likely due to the activation of AMPK. This is supported by previous findings showing that AMPK phosphorylation stimulates transcription factors involved in mitochondrial biogenesis and functionality [31,32] as well as HSL activation, lipid breakdown and β-oxidation pathway [33,34].

**Fig 5:**
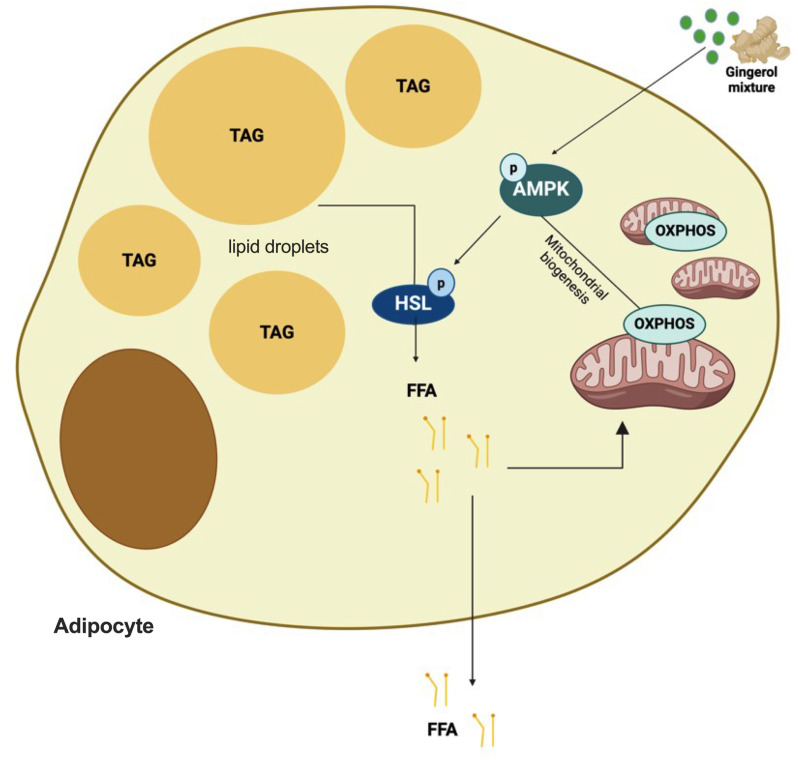
Cellular mechanisms of ginger phenolic compounds on adipocytes. A gingerol mixture (comprising 6-, 8-, and 10-gingerol, as well as 6-, 8-, and 10-shogaol) stimulates the activation of AMP-activated protein kinase (AMPK), which leads to enhanced mitochondrial biogenesis and oxidative phosphorylation (OXPHOS). Simultaneously, AMPK activation phosphorylates hormone-sensitive lipase (HSL), which catalyzes the breakdown of triglycerides (TAG) into free fatty acids (FFA). The liberated FFAs can be utilized in mitochondrial oxidation to support energy production or released into the culture medium.

Additionally, the enhanced reduction of lipid droplets observed with the gingerol mixture, in comparison to AICAR, might indicate a synergistic effect from various signaling pathways activated by gingerols. AICAR functions as a quick and temporary AMPK activator and serves as a benchmark to confirm AMPK signaling activation; therefore, adipocytes were stimulated with AICAR for 60 minutes. Conversely, gingerol treatment likely activates both AMPK-dependent and independent pathways, necessitating an extended incubation period of 48 hours to fully harness the activities of gingerols in adipocytes. The results imply that gingerols function as multi-target agents with prolonged effects on the lipid metabolism of adipocytes (Fig 5). Together, these findings support a growing role for gingerols as modulators of energy metabolism and mitochondrial function through AMPK-dependent pathways. These findings underscore the potential of gingerols as multi-target therapeutic candidates for metabolic and cardiovascular diseases.

Dietary phenolic compounds, including resveratrol, epigallocatechins, berberine, curcumin, quercetin, and genistein, have been shown to modulate AMPK, promoting oxidative metabolism and reducing inflammation [35]. For instance, Abelmoschus esculentus (okra), which contains high levels of quercetin, enhances glucose and lipid profiles in diabetic rats by increasing AMPK activation while reducing PEPCK and HSL expression [36]. Similarly, adlay polyphenols extracted from Coix lacryma-jobi L. reduce lipid accumulation and improve lipid metabolism via the p-AMPK/p-ACC pathway in FFA-treated HepG2 cells, and lower body and liver weight, hepatic triglycerides, cholesterol, and serum glucose in high-fat diet-fed mice [37]. Hawthorn polyphenol microcapsules enhance skeletal muscle metabolism and antioxidant capacity in exercise-fatigued mice by activating AMPK and suppressing the NF-κB pathway [38]. Other polyphenols, such as honokiol and ferulic acid, have been shown to protect mitochondrial integrity and promote autophagy in models of acute kidney and liver injury through AMPK activation [39,40].Additionally, pecan polyphenols improve AMPK activity in the skeletal muscle of obese mice, enhancing mitochondrial function, metabolic flexibility, and insulin sensitivity [41]. Chaya leaf extracts, rich in polyphenols, further support this trend by promoting mitochondrial bioenergetics and fatty acid oxidation in myotubes and hepatocytes via AMPK signaling [42].These lines of evidence demonstrate that various plant polyphenols can activate AMPK, improving metabolic parameters. However, the specific effects of ginger-derived phenolic compounds—particularly gingerols and shogaols— on AMPK activity remain less explored. The present findings suggest that the gingerol–shogaol mixture modulates adipocyte metabolism at least in part, through AMPK activation, highlighting the potential of ginger root as a non-pharmacological approach to energy balance modulation.

Our findings underscore the potential of a gingerol mixture as promising coadjuvant in the treatment of metabolic diseases. By improving mitochondrial function, enhancing lipolysis, and reducing lipid accumulation, the gingerol mixture could play a crucial role in restoring metabolic homeostasis in adipose tissue. These results are particularly significant given the central role that mitochondrial dysfunction and lipid dysregulation play in obesity-related metabolic disorders (Fig 5). Thus, gingerol mixture represents a novel therapeutic strategy that could complement existing interventions for obesity management, potentially mitigating some of the adverse metabolic effects associated with this condition. The translatability of the present study depends on whether the dose of gingerols used to stimulate adipocytes can be achieved through a reasonable dietary intake of ginger. In this line, Suzanna M. Zick and colleagues evaluated the pharmacokinetic profile of 6-, 8-, and 10-gingerol, as well as 6-shogaol and their conjugate metabolites administered orally to healthy human volunteers. After ingestion of a 2g ginger extract standardized to 5% gingerols, they observed a plasma concentration of 1.69 μg/mL for 6-gingerol, 0.23 μg/mL for 8-gingerol, 0.53 μg/mL for 10-gingerol, and 0.15 μg/mL for 6-shogaol 30 minutes after oral dosing, reaching their Tmax between 45 and 120 min [43].We treated adipocytes with 6 μg/mL of the gingerol mixture. This concentration could be achieved by consuming 4 g of a ginger extract as in Zick et al. However, gingerols and shogaols may reach higher concentrations in the interstitial fluid within target tissue compared to serum [44].Thus, it is likely that the dose of 2 g ginger extract (or 5 g of fresh ginger root) could achieve an interstitial gingerol concentration similar to that used in this study.

Despite the promising outcomes, this study has certain limitations that must be considered. The in vitro nature of the experiments restricts the generalizability of our findings to clinical settings. The study was performed only in inguinal (subcutaneous) adipocytes and not visceral. Since the aim of the study was to explore the mechanisms by which gingerols enhance mitochondrial activity, the rationale was that subcutaneous adipocytes have greater mitochondrial abundance and respiratory capacity than visceral adipocytes, which are more lipolytic but possess lower mitochondrial function [45].While this model allowed us to study gingerol-induced AMPK activation and its impact on bioenergetics and lipolysis under functional conditions, future studies should address visceral adipocytes, particularly given their metabolic relevance in obesity. In prior work, we demonstrated that adipocytes derived from obese rats exhibit impaired mitochondrial function due to epigenetic alterations driven by the obesogenic environment [46].Therefore, assessing gingerols in adipocytes from obese models is a logical next step. We also recognize the relevance of age-related changes; however, comparing adipocytes from young and old animals presents methodological challenges, as aging reduces the number and differentiation potential of adipose-derived mesenchymal stem cells. Despite this limitation, we are exploring strategies to address this question in future research. Further research, particularly in vivo studies, is required to confirm the effects of the gingerol mixture on mitochondrial function and lipid metabolism in human subjects. Moreover, elucidating the exact molecular mechanisms through which gingerols activate AMPK and other metabolic pathways would provide valuable insights into their therapeutic potential. Future studies should also assess the long-term impact of gingerol supplementation in obesity models to better understand its efficacy and safety as an adjunct treatment.

## Conclusion

The gingerol mixture regulates energy metabolism in adipocytes by enhancing AMPK-mediated mitochondrial activity and promoting lipolysis, thereby preventing adipocyte hypertrophy and the energetic disturbances characteristic of obesity. Characterizing the cellular mechanisms involved in the beneficial effects of dietary compounds offers novel insights into nutritional intervention strategies aimed at preventing and managing obesity and related metabolic diseases.

## Supporting information

S1_raw_images Raw images of immunoblots. (PDF)
