# Supplementary material for: A mixture of ginger phenolic compounds enhances mitochondrial function, activates AMPK, and reduces lipid accumulation in adipocytes
Source: PLoS One. 2025 Jun 27;20(6):e0326690. doi: 10.1371/journal.pone.0326690 (PMC12204587; doi:10.1371/journal.pone.0326690)

AMPK
Figure 3C

Lane
1-MWM
2-Veh 1
3-Veh 2
4-Veh 3
5-Ging 1
6-Ging 2
7-Ging 3
8-AICAR 1
9-AICAR 2
10-AICAR 3

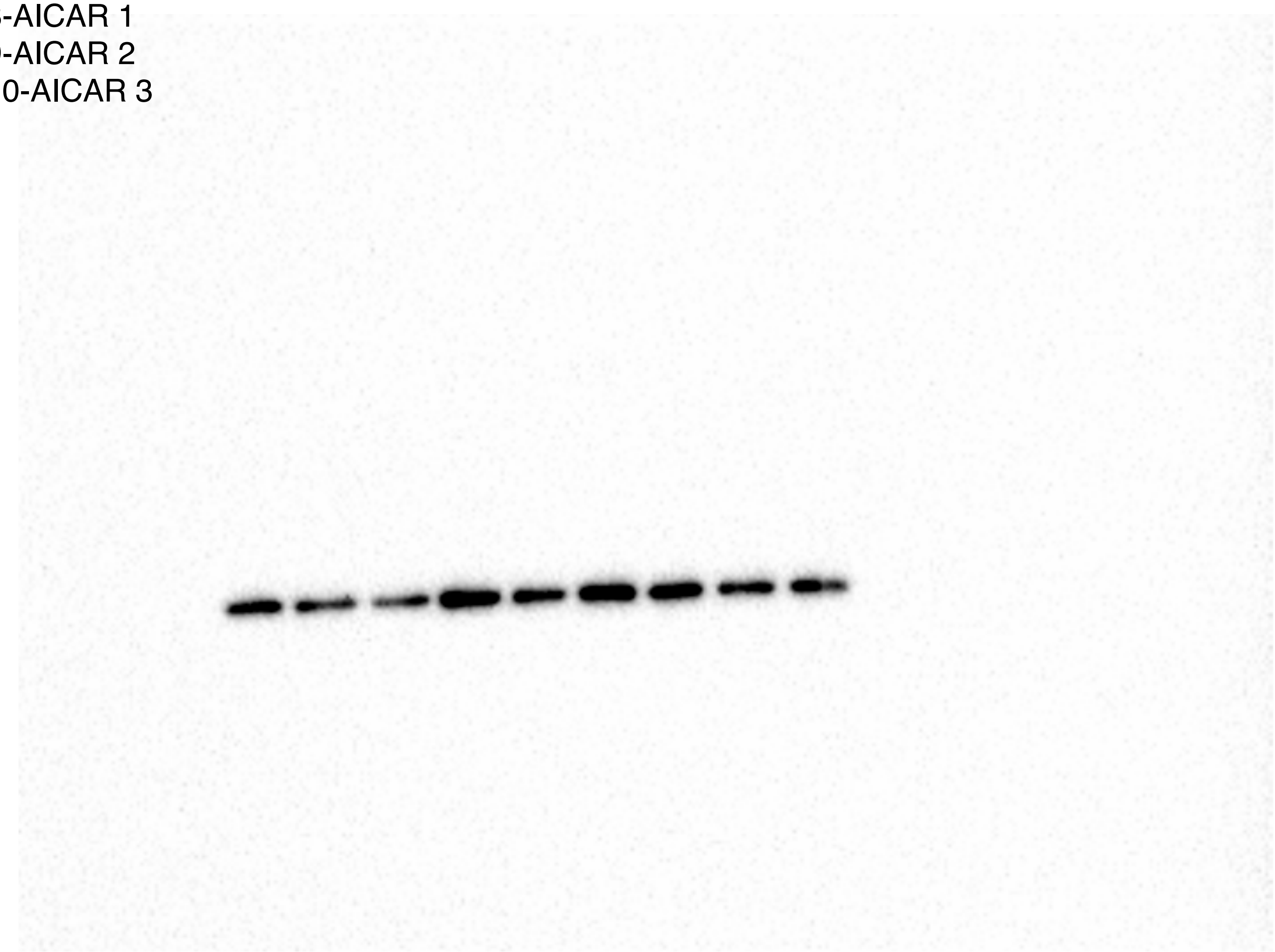

p-AMPK
Figure 3C

Lane
1-MWM
2-Veh 1
3-Veh 2
4-Veh 3
5-Ging 1
6-Ging 2
7-Ging 3
8-AICAR 1
9-AICAR 2
10-AICAR 3

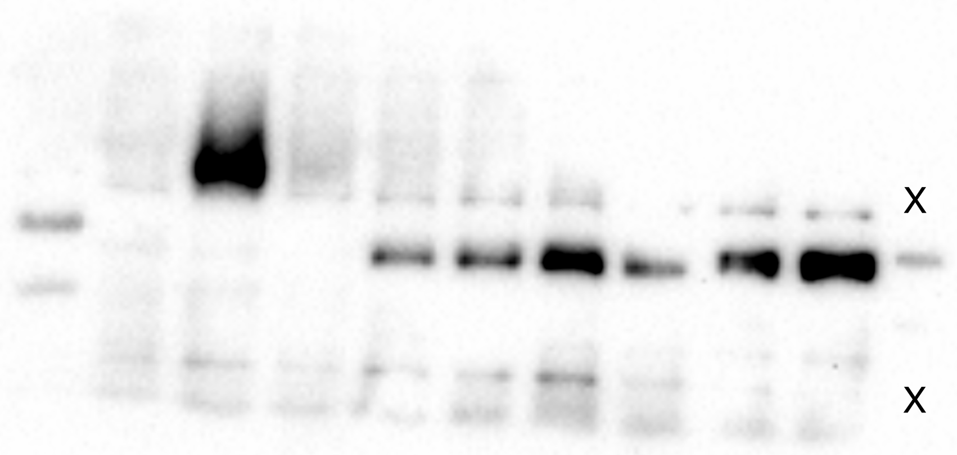

OXPHOS
Figure 3C

Lane
1-MWM
2-Veh 1
3-Veh 2
4-Veh 3
5-Ging 1
6-Ging 2
7-Ging 3
8-AICAR 1
9-AICAR 2
10-AICAR 3

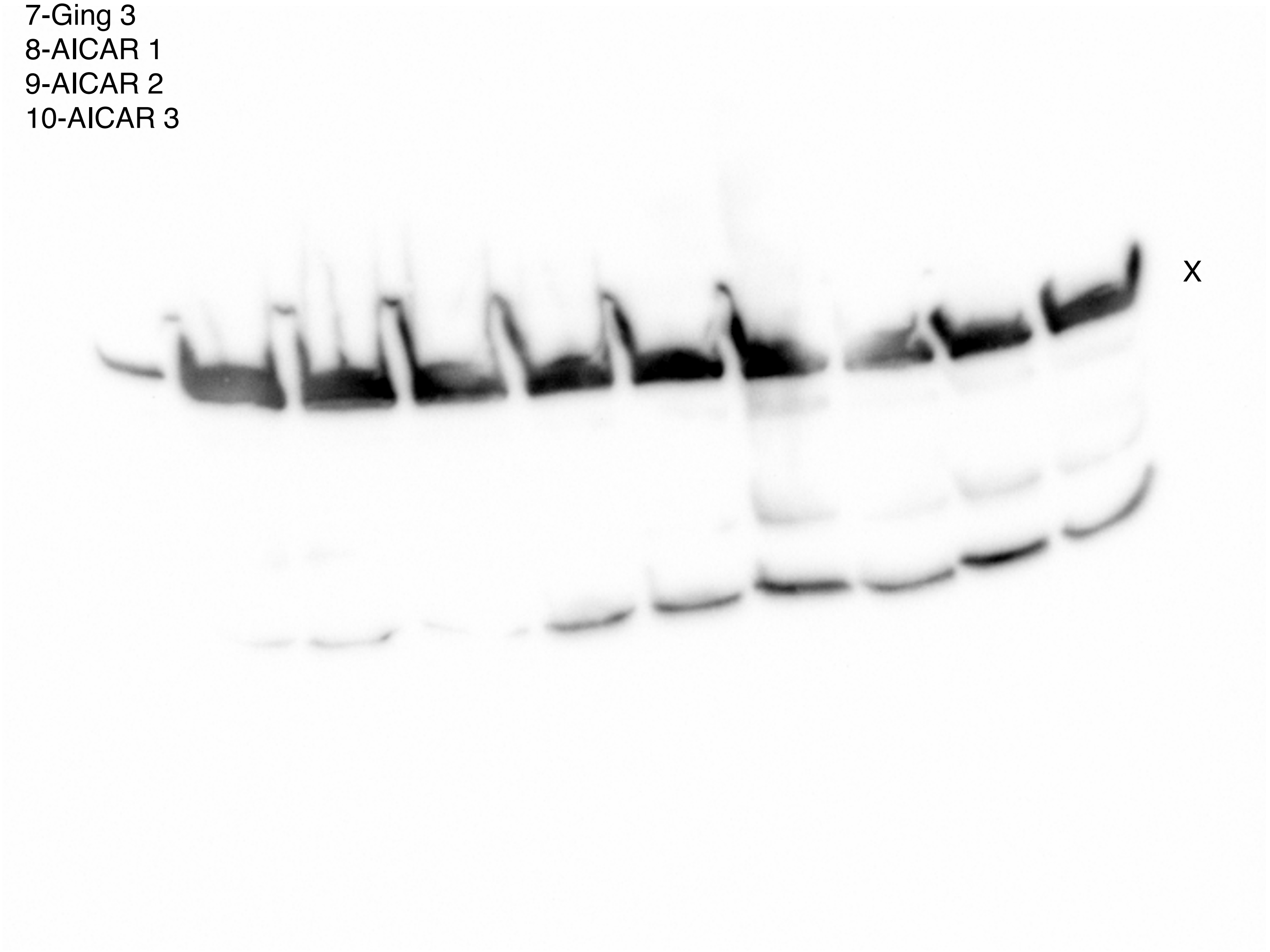

GAPDH
Figure 3C

Lane
1-MWM
2-Veh 1
3-Veh 2
4-Veh 3
5-Ging 1
6-Ging 2
7-Ging 3
8-AICAR 1
9-AICAR 2
10-AICAR 3

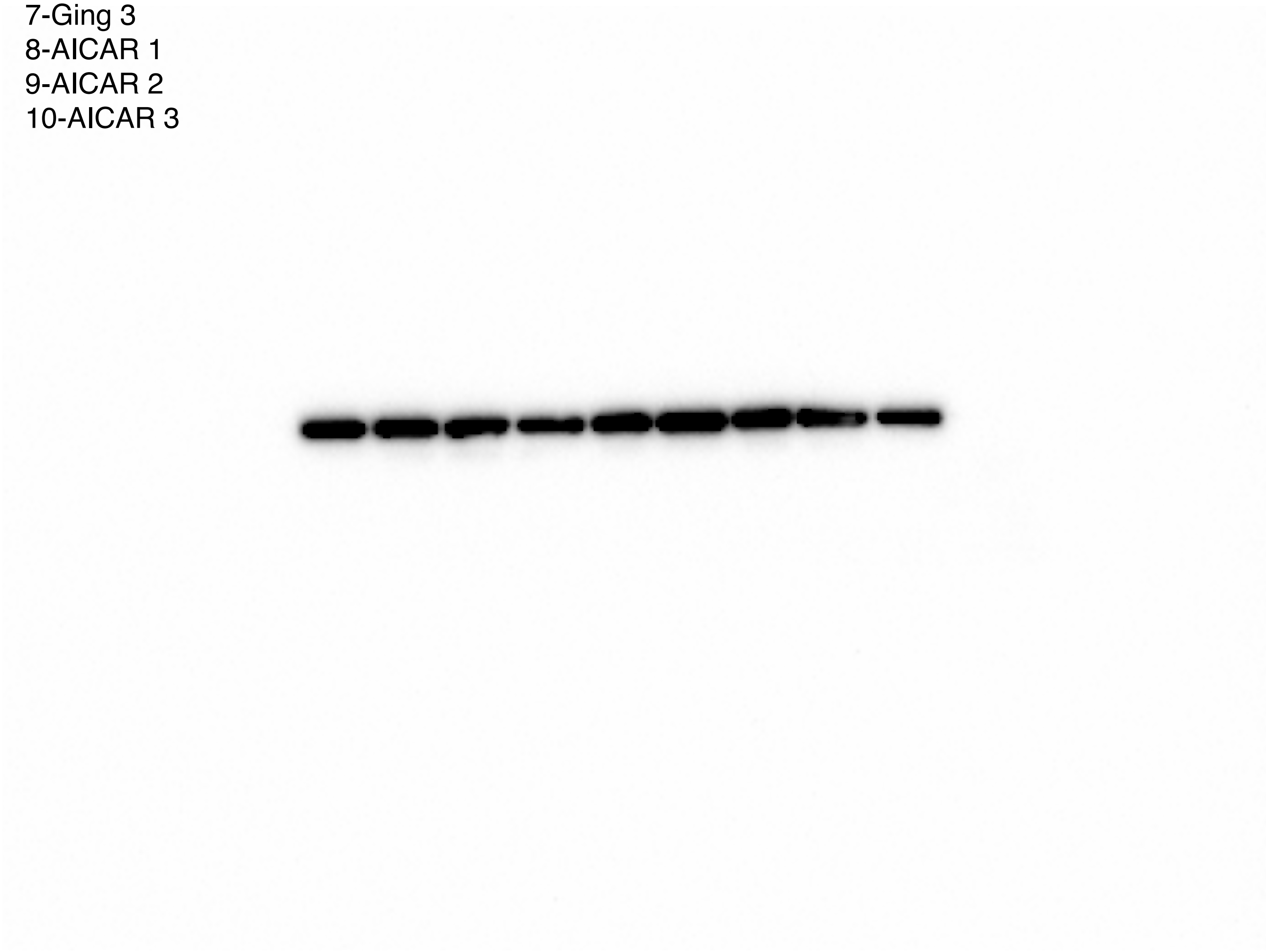

p-HSL
Figure 4C

Lane
1-MWM
2-Veh 1
3-Veh 2
4-Veh 3
5-Ging 1
6-Ging 2
7-Ging 3
8-AICAR 1
9-AICAR 2
10-AICAR 3

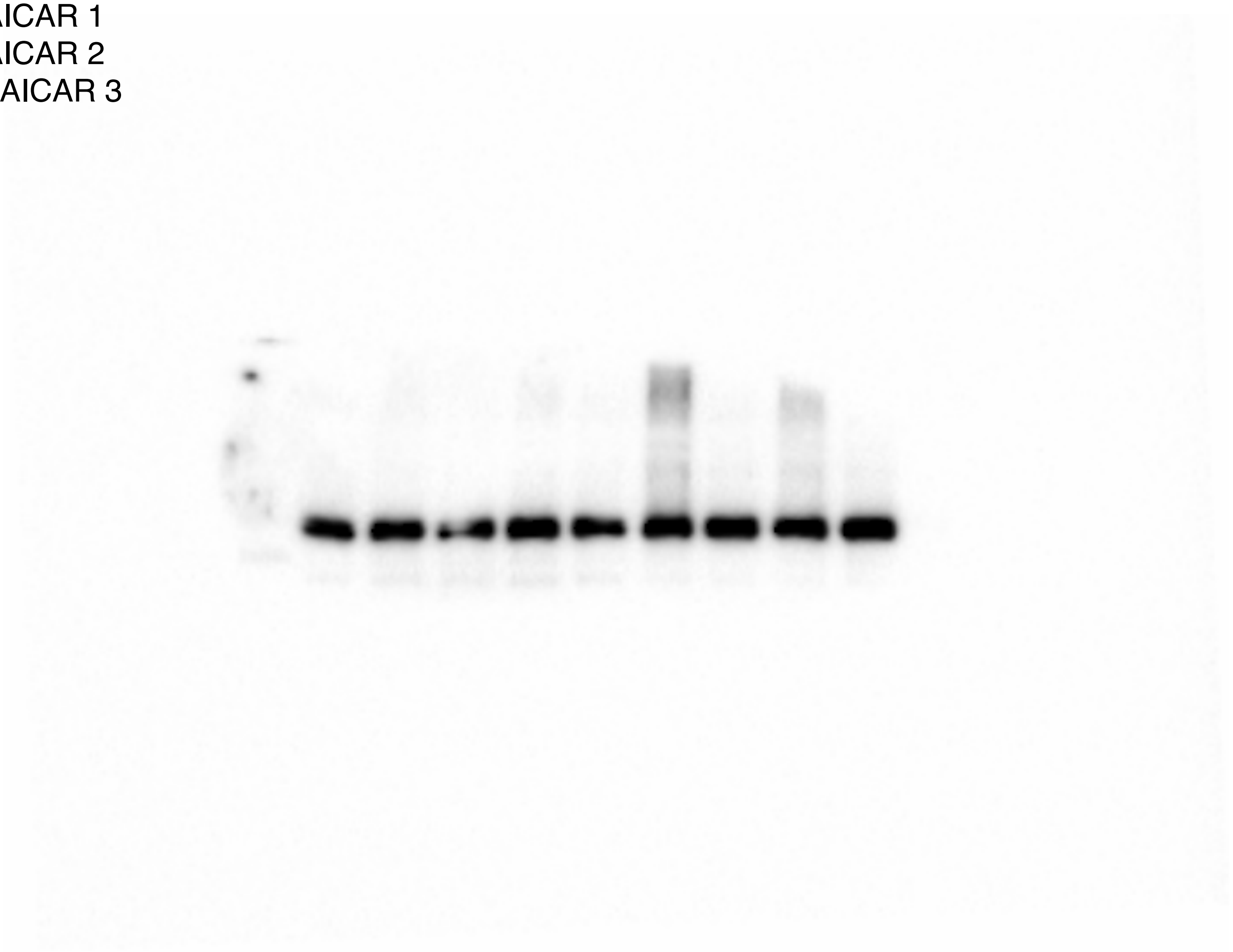

GAPDH
Figure 4C

Lane
1-MWM
2-Veh 1
3-Veh 2
4-Veh 3
5-Ging 1
6-Ging 2
7-Ging 3
8-AICAR 1
9-AICAR 2
10-AICAR 3

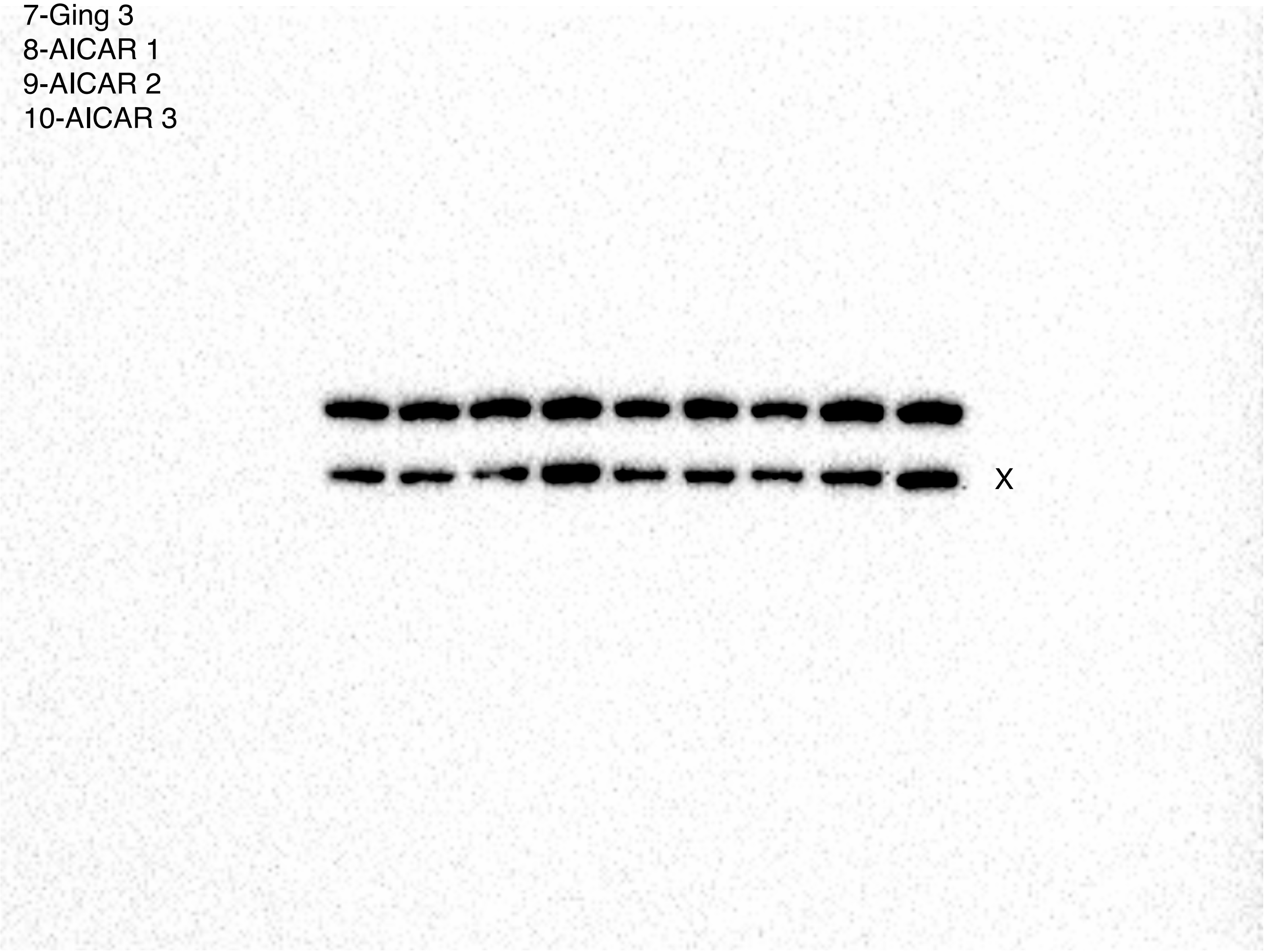

Supplement: S1_raw_images — (PDF) [file pone.0326690.s001.pdf]
